# Salinity Gradient of the Baltic Sea Limits the Reproduction and Population Expansion of the Newly Invaded Comb Jelly *Mnemiopsis leidyi*

**DOI:** 10.1371/journal.pone.0024065

**Published:** 2011-08-26

**Authors:** Cornelia Jaspers, Lene Friis Møller, Thomas Kiørboe

**Affiliations:** 1 National Institute of Aquatic Resources, Technical University of Denmark, Charlottenlund, Denmark; 2 Department of Marine Ecology – Kristineberg, University of Gothenburg, Fiskebäckskil, Sweden; Institute of Marine Research, Norway

## Abstract

The recent invasion of the comb jelly *Mnemiopsis leidyi* into northern European waters is of major public and scientific concern. One of the key features making *M. leidyi* a successful invader is its high fecundity combined with fast growth rates. However, little is known about physiological limitations to its reproduction and consequent possible abiotic restrictions to its dispersal. To evaluate the invasion potential of *M. leidyi* into the brackish Baltic Sea we studied in situ egg production rates in different regions and at different salinities in the laboratory, representing the salinity gradient of the Baltic Sea. During October 2009 *M. leidyi* actively reproduced over large areas of the Baltic Sea. Egg production rates scaled with animal size but decreased significantly with decreasing salinity, both in the field (7–29) and in laboratory experiments (6–33). Temperature and zooplankton, i.e. food abundance, could not explain the observed differences. Reproduction rates at conditions representing the Kattegat, south western and central Baltic Sea, respectively, were 2.8 fold higher at the highest salinities (33 and 25) than at intermediate salinities (10 and 15) and 21 times higher compared from intermediate to the lowest salinity tested (6). Higher salinity areas such as the Kattegat, and to a lower extent the south western Baltic, seem to act as source regions for the *M. leidyi* population in the central Baltic Sea where a self-sustaining population, due to the low salinity, cannot be maintained.

## Introduction

Invasive species in marine environments have gained public and scientific attention due to their documented direct and cascading effects, once successfully established, on ecosystems as well as biodiversity [1]. Ecosystems disturbed by, for example, eutrophication or overfishing are especially vulnerable to invasions [2]. The ctenophore *Mnemiopsis leidyi*, native to the east coast of the Americas, is such a successful invasive species as documented after its introduction into the Black Sea in the 1980's [3]. In 2005, *M. leidyi* was first sighted in northern European waters [4] and has since spread over large areas such as the North Sea [5], all Danish waters [6], as well as the western [7] and central Baltic Sea [8], [9]. The invasion success of *M. leidyi* is partly due to its high reproduction capacity, being a hermaphrodite with a daily production of up to 14,000 eggs ind^−1^ [10].

**Figure 1:**
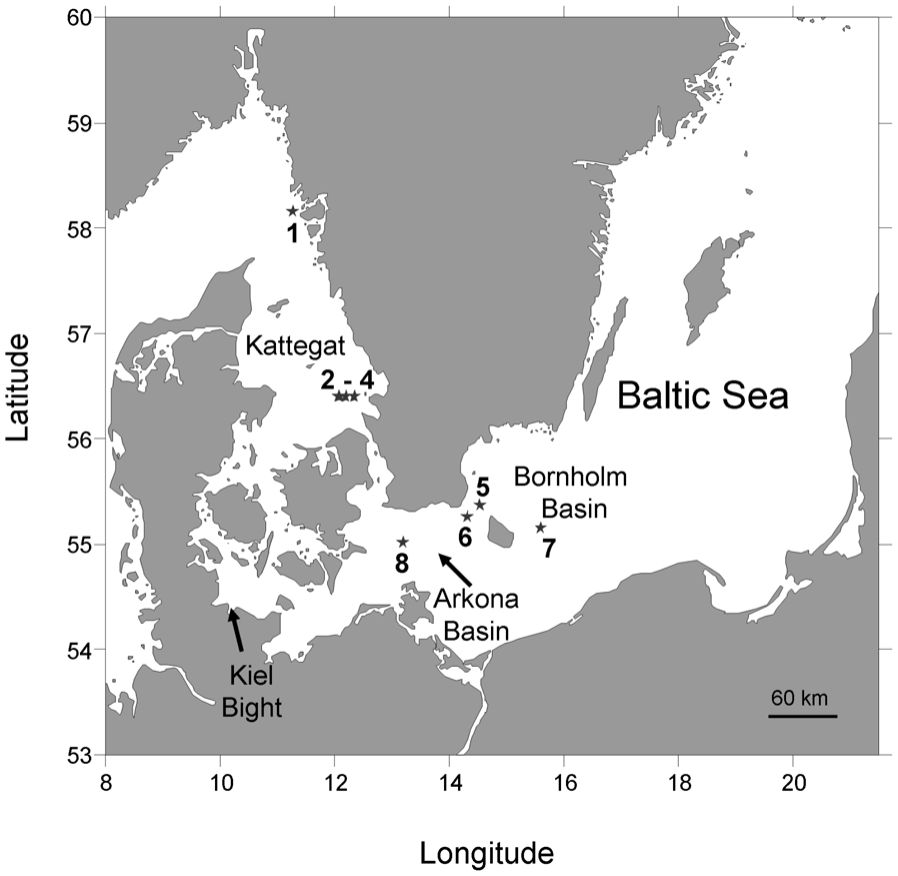
Investigation area. In situ *Mnemiopsis leidyi* egg production experiments were performed in the Kattegat (Stations 1–4, high salinity) and central Baltic Sea (Stations 5–8, low salinity) in October 2009. Locality or area names are specified as used throughout the manuscript. Station 2 to 4 comprise a short transect.

To better predict how *M. leidyi* will expand into, and potentially impact, the Baltic Sea it is important to understand how environmental characteristics affect its reproduction capacity. Generally, the distribution of marine organisms are governed by their physiological tolerance to biotic and abiotic factors and the demographics will be restricted by their tolerance limits [11]. In the Baltic Sea, the salinity gradient has been shown to influence invasive mesozooplankton species with higher species number in higher salinity waters [12] whereas in the western Mediterranean Sea mass occurrences of invasive jellyfish have been documented to be directly correlated to temperature [13]. In *M. leidyi*, reproduction has been shown to vary with temperature and food availability [14]–[19], but the dependency on salinity is unknown. The Baltic Sea is one of the largest brackish water bodies in the world and characterized by strong vertical and horizontal salinity gradients. The surface salinity ranges from around 4 in the north-east, to 6–8 in the central Baltic, 15–25 in the south-west [20], and increases to >18–33 in the connecting Danish Straits and the Kattegat (Fig. 1). The question arises whether blooms, as observed in other invaded and native habitats [3], [15], can be expected for the entire Baltic Sea or if salinity will restrict the population expansion.

The aim of this work was to evaluate effects of salinity on egg production by *M. leidyi* in the newly invaded Baltic Sea. We examined in situ egg production rates of *M. leidyi* during its seasonal abundance peak in high and low salinity areas of the Baltic Sea and carried out laboratory experiments to test the effect of salinity on egg production rates. We can show that salinity has a strong impact on reproduction rates and seems to limit the establishment of a year round, self-sustained *M. leidyi* population in the central Baltic Sea.

## Materials and Methods

### Ethics statement

No specific permits were required for the described field and laboratory studies. The locations visited are not privately-owned or protected and the field studies did not involve endangered or protected species.

### Study area

The overall hydrography of the Baltic Sea is governed by large fresh water input in the north eastern parts that is compensated by surface water outflow through the Danish Straits and subsequent deep water inflow, with detailed dynamics governed by meteorological forces [20]. Our investigation covered the Bornholm and north Arkona Basin (hereafter referred to as central Baltic) which has a low surface salinity (7–9) and the Swedish west coast (Kattegat) which is characterized by a much higher surface salinity (21–29).

### Field investigation

In situ egg production rates of *M. leidyi* were measured at 8 stations in the Kattegat and central Baltic Sea (Fig. 1) during 12–21 October 2009 onboard R/V Skagerak (University of Gothenburg). Vertical profiles of salinity and temperature were measured using a Seabird 9/11 CTD. Assessment of food availability for *M. leidyi* was based on zooplankton abundances. Zooplankton was collected in 10 m depth strata with a HYDRO-BIOS© MultiNet sampler (0.25 m^2^ net opening) equipped with 90-µm nets and preserved in 2% acidified Lugol solution. Zooplankton sizes were corrected for shrinkage using a correction factor of 1.17 for chitineous and 1.22 for gelatinous zooplankton [21]. Their biomass was estimated from the average sizes of developmental stages for all copepod species and the average sizes for nauplii, cladocerans and other zooplankton groups, applying species specific regressions if applicable [following 22]. Dry weight or ash free dry weight were converted to carbon using conversion factors of 0.4 [23] and 0.45 [24], respectively.

Egg production experiments were performed at 4 stations (St.) in each of the Kattegat (St. 1–4) and the central Baltic Sea (St. 5–8), with 52 and 47 individual experiments respectively (Fig. 1). Station 2 egg production rates were measured twice at noon for 2 subsequent days; at all other stations experiments were performed only once. Station 2 to 4 comprised a short transect. *M. leidyi* were caught with a black, 2 mm mesh size, 1.7 m^2^ and 5 m long ring net with a 10 L non filtering cod end. Animals were collected in the upper 10 m of the water column. The cod end was opened into a 20 L bucket pre-filled with incubation water (from 5 m depth) and immediately transferred into a temperature controlled room (12.5±1.3°C). Incubation container volumes ranged from 1 to 13.5 L. No effect of container size on egg production was observed, neither for 2.8 cm animals in 1 and 13.5 L (t = 0.31, p = 0.765, df = 6), nor for size-specific egg production rates in 1, 2 and 13.5 L containers in the Kattegat (F = 1.067, p = 0.352, df = 2). Experiments started within 1 hour after collection by gently transferring, into individual containers, actively swimming *M. leidyi* that showed no signs of damage. Egg production was measured over 24 h including one full night and following the natural light regime, at constant temperature (12.5±1.3°C) and irrespective of collection temperature (8 to 12°C). At termination, *M. leidyi* were removed and measured to the nearest mm (oral-aboral length). Eggs were concentrated by reverse filtration (45 µm), preserved in 2% acidified Lugol solution and enumerated within two weeks. Sizes were converted by use of the oral-aboral length (L, mm) to volume (V, ml) regression: V = 0.0226×L^1.72^ [25], and rates presented as volume-specific egg production (eggs mL *Mnemiopsis* ^−1^ day^−1^).

### Laboratory experiments

To confirm in situ observations, laboratory controlled salinity-dependent egg production experiments were conducted at the Sven Lovén Centre for Marine Sciences, Sweden in June 2011. *M. leidyi* originated from laboratory cultures kept at a salinity of 33 that were originally caught in the Kattegat (58°15′N 11°24′E). Experimental animals were raised to a standard size of 15 mm from a cohort spawned in April 2011. Animals were acclimated over 5 days via step wise dilution to the target salinities (6, 10 15, 25 and 33) and kept at those conditions for at least 7 days before the start of the experiments. Animals were fed ad libitum with *Acartia tonsa* from laboratory cultures and their carbon content was estimated from literature regressions [following 22]. Copepods were acclimated to the different salinities for at least 24 h before the start of the experiments. Individual egg production was measured in filtered seawater during 24 h in 7.5 L incubation container after 12 h food acclimatization at a mean copepod prey concentration of 75 µg Carbon L^−1^. Prey concentrations at the end of preconditioning did not vary between treatments (p = 0.23). Eggs were counted and animals shifted to fresh water every 12 h to prevent egg cannibalism, even though *M. leidyi* does not cannibalize their eggs [26] but have been shown to intensively prey on their larvae >5 mm [27]. Egg production rates are presented as individual rates 24 h^−1^. Egg production experiments always started at 7 pm and the overall experimental temperature was 18.8±0.4°C.

### Statistical analyses

Statistical analyses were conducted in R 2.13.0 (www.r-project.org/) using a significant level of 0.05. Size dependent egg production experiments were analyzed using power regression analyses on raw data. A separate slopes model was used to test for differences between slopes on log (x+1) transformed data. Laboratory egg production rates were square root transformed to meet normality assumptions before 1-way ANOVA and subsequent equality of variance and homogeneity tests were performed. Student Newman Keuls post hoc test was used to detect significant groupings.

## Results

### Hydrography

In October 2009 we observed a typical situation with marked surface salinity differences between high salinity stations in the Kattegat (25±3, average in upper 10 m) compared to low salinity waters in the central Baltic (7.8±0.3). Surface temperatures in the central Baltic ranged from 8.4°C at station 5 to 10.7°C at station 7 and 8 and 11.3±0.8°C at all Kattegat stations. Across all stations the surface temperature averaged 11±1.2°C. The mixing depth of the surface waters differed between the two areas with a shallower upper mixed layer in the Kattegat between 10 m and 20 m, compared to 35 m for all central Baltic stations.

### Egg production in the field

**Figure 2:**
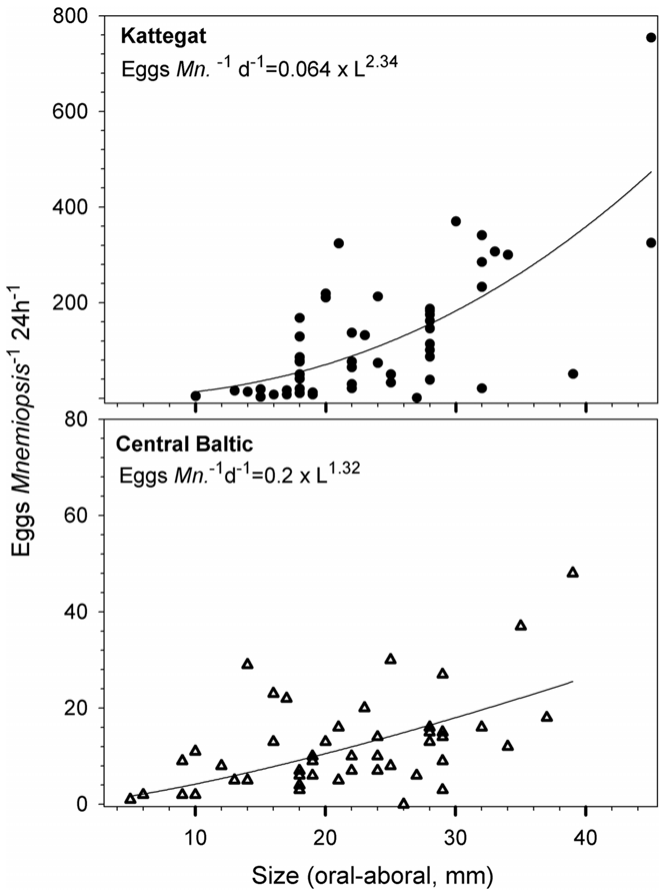
In situ size dependent reproduction rates of the invasive comb jelly *Mnemiopsis leidyi*, northern Europe. The egg production differed significantly between the two investigation areas (F = 12.28, p = 0.0007, df = 95) with higher rates in the Kattegat (n = 52) than in the central Baltic Sea (n = 47), 12.5±1.3°C, October 2009. Per capita egg production scaled significantly with length (oral aboral, mm). The observed production for a 15.1 mm standard animal used during salinity-dependent laboratory experiments is 37 versus 7 eggs *Mnemiopsis* ^−1^ d^−1^ in the Kattegat and central Baltic, respectively.

We found no difference in volume-specific egg production rates between low and high in situ temperatures in the central Baltic (F = 2.28, p = 0.116, df = 2). Per capita egg production was a power function of body length, with exponents of 2.3±0.3 for the high salinity Kattegat compared to 1.3±0.4 for the low salinity central Baltic (Fig. 2). Animals that originated from the Kattegat showed a factor 10 higher reproduction rate than *M. leidyi* from the central Baltic (Fig. 2).

**Figure 3:**
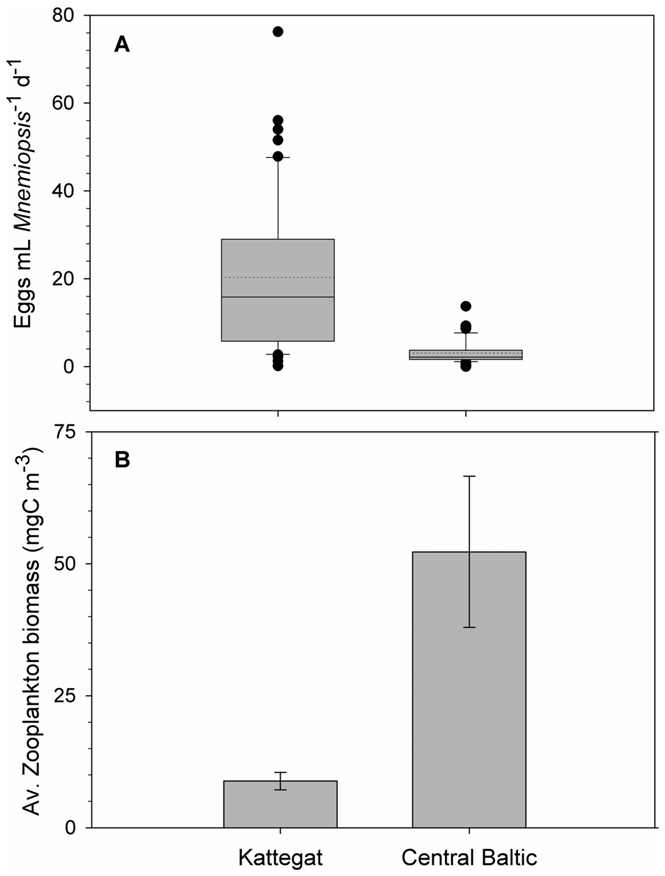
Field observations of *Mnemiopsis leidyi* standardized in situ egg production and average zooplankton food concentrations. **A**) Box-whisker plot of standardized egg production rates of *M. leidyi* in the Kattegat (n = 52) and central Baltic Sea (n = 47) with median indicated by solid line, mean presented as dotted line, 75 and 25 percentiles stated in the box; 90 and 10 percentiles indicated by error bars. Standardized egg production was significantly different between the two regions (t = 6.77, p<0.0001, df = 97) with 20±17 versus 3±2.5 eggs mL *Mnemiopsis* ^−1^ d^−1^ in the Kattegat and central Baltic, respectively. **B**) Zooplankton biomass as a measure of potential *M. leidyi* food availability. Zooplankton biomass was 6 times higher in the central Baltic compared to the Kattegat (t = 3.012, p = 0.024, df = 6). Bars present average biomasses (±SE) for the same stations where egg production was investigated.

Volume-specific production rates also differed significantly between the two regions. The Kattegat showed a nearly 7 times higher average specific production of 20±17 compared to 3±2.5 eggs mL *Mnemiopsis* ^−1^ d^−1^ in the central Baltic (Fig. 3A).

In both areas, the major part of the zooplankton biomass was comprised of copepods. The potential food quality for *M. leidyi* was therefore similar between the two regions, whereas the zooplankton biomass in the central Baltic was 6 times higher than in the Kattegat (Fig. 3B).

### Laboratory salinity-dependent egg production rates

**Figure 4:**
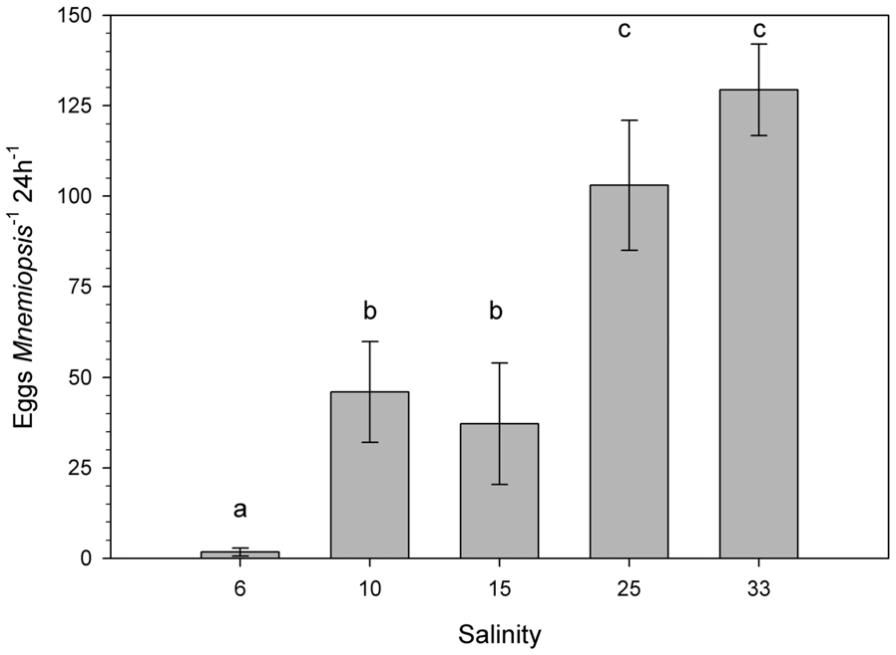
*Mnemiopsis leidyi* egg production at salinities representative for different hydrographic regimes of the Baltic Sea. Laboratory reared animals (mean oral aboral length 15.1±1.8 mm, n = 4–5) were individually incubated and mean egg production per salinity is stated 24 h^−1^ as bars ±SE. Egg production rates differed significantly (F = 41.33, p<0.0001, df = 22) with salinity forming 3 clusters of 33/25, 15/10 and 6 (Student-Newman-Keuls post hoc test with significant groups indicated by letters a–c).

The size of individuals used in the 5 salinity treatments did not differ significantly and the overall average size was 15.1±1.8 mm (oral aboral length) (Table 1). Egg production was significantly affected by salinity (F = 41.33, p<0.0001, df = 22 see Table 1, Fig. 4). Highest egg production rates were obtained at the highest salinities tested (25 and 33) with an average of 40 eggs mL *Mnemiopsis* ^−1^ d^−1^. At intermediate salinities of 15 and 10, egg production was significantly lower and the lowest egg production rates were observed at the lowest salinity of 6 with 0.9 eggs mL *Mnemiopsis* ^−1^ d^−1^.

**Table 1:** Average *Mnemiopsis leidyi* sizes and reproduction rates from laboratory salinity-dependent production experiments in Sweden, June 2011.

Salinity	Oral aboral length (mm)		Egg production (Ind^−1^ d^−1^)		Replicate
	*Average*	*SD*	*Average*	*SD*	*n*
**6**	13.7	1.5	2	2.2	4
**10**	14.8	1.8	46	31.2	5
**15**	15.6	2.0	37	37.5	5
**25**	16.3	1.1	103	40.2	5
**33**	14.6	2.1	129	28.3	5

The overall animal sizes used was 15.1±1.8 mm and did not differ between salinity treatments (F = 0.855, p = 0.365, df = 22).

## Discussion

Earlier reviews [e.g. 14], [15] emphasized the importance of temperature, zooplankton and predators in determining the *Mnemiopsis leidyi* population size without considering the effect of salinity. Here we have demonstrated both from in situ observations and in laboratory experiments that the reproduction rate of the invasive ctenophore *M. leidyi* is highly sensitive to the range of salinities found in the newly invaded Baltic Sea area.

*M. leidyi* had tenfold higher egg production rates in the high salinity Kattegat area compared to the low salinity central Baltic Sea. Food availability has been reported to strongly influence egg production in *M. leidyi* [e.g. 19], [28] but in situ reproduction rates were highest in areas with relatively lower food availability (Kattegat) and hence the observed effect of salinity in the field data is conservative.

Salinity-dependent egg production rates in the laboratory, where food, temperature and sizes were kept constant, confirmed our field observations. We found almost no reproduction at a salinity of 6, representative for the conditions in the central Baltic, intermediate reproduction at salinities of 10 to 15 characteristic for the south western Baltic Sea, and highest rates at salinities of 25 to 33 as observed in the Kattegat. Our results suggest that salinity should be considered as an important explanatory variable in determining in situ reproduction rates. If we compare our results with published size-specific production rates from native habitats in the US, a large scatter within the same temperature ranges is obvious [e.g.15]. However, the average egg production rate at 12 to 16.5°C in the US is similar to high salinity in situ reproduction rates measured in the Kattegat (12.5±1.3°C), viz. 20 eggs mL *Mnemiopsis* ^−1^ d^−1^.

### Distribution

After the first sighting of *M. leidyi* in 2005 [4], it has rapidly become abundant in northern European waters with the highest densities in the Kattegat and south western Baltic especially during summer [6], [7], [29]. So far, *M. leidyi* is generally absent from the central Baltic Sea during summer, e.g., the Bornholm Basin, but appears in these low salinity areas in low abundances from autumn to spring [8]. In the south western Baltic the *M. leidyi* abundances peak in August, corresponding to the reproduction peak as shown from analysis of population size structures [7]. The lack of *M. leidyi* in the central Baltic during summer may therefore indicate poor reproduction and survival. Our study shows that *M. leidyi* is hardly reproducing at the low salinities found in the central Baltic which might explanation their low abundances. It seems that the appearance of *M. leidyi* in this area must be due to drift of individuals from higher salinity source areas [8].

Drift model studies and abundance observations have suggested that the southern Kattegat and south western Baltic Sea are source areas for the population of the jellyfish *Aurelia aurita* in the central Baltic Sea, e.g., the Bornholm Basin [30]. Drift models estimate that it takes about 2 months for animals recruited in the Danish Straits and the Kiel Bight to reach the Bornholm Basin [30]. Consistently, the *M. leidyi* in the Bornholm Basin appears ca. 2 months [8] after its peak occurrence in the south western Baltic [7]. Further evidence for drift recruitment is that both species early in the season are found mainly in the deeper, higher salinity, depth strata in water of more western origin [8], [30]. Even though the salinity at depth is higher, ranging between 7–14 at the maximum abundance position of *M. leidyi* around the halocline [9], [31], the year round low temperatures [8] probably constrain their reproduction at this depth due to very low feeding rates [32].

Our laboratory results suggest that the south western Baltic Sea may be a source area for the central Baltic *M. leidyi* autumn population. In the Kiel Bight area the reproduction peaks at salinities above 15 and high summer temperatures (>14°C) as concluded from population size structure analysis [7]. In this area, animals are present the whole year, which indicates that a self-sustained subpopulation has established in the intermediate saline waters of the south western Baltic Sea [7].

### Conclusion

The documented predatory impact of *M. leidyi* has lead to the concern of its range expansion and local population establishment especially in the central Baltic. The Bornholm Basin is of special interest since this is the most important cod spawning ground in the central Baltic [33]. Previous work has shown that *M. leidyi* does not constitute a direct threat to cod eggs and larvae in this area as a predator [32], although it may compete with larval cod for zooplankton prey. Here we demonstrate that the reproduction rates of the invasive ctenophore *M. leidyi* are considerably reduced under the low salinities that are characteristic of the central Baltic Sea. Hence, salinity acts as a bottle neck for the population expansions in this newly invaded area. Higher salinity areas such as the Kattegat, and to a lower extent the south western Baltic, seem to act as source regions for the *M. leidyi* population in the central Baltic Sea where a self-sustaining population, due to the low salinity, cannot be maintained. Hence, both in terms of direct and indirect effects *M. leidyi* is unlikely to become a threat to early life stages of cod in the central Baltic.
